# Computational Study to Determine When to Initiate and Alternate Therapy in HIV Infection

**DOI:** 10.1155/2014/472869

**Published:** 2014-05-11

**Authors:** Matthias Haering, Andreas Hördt, Michael Meyer-Hermann, Esteban A. Hernandez-Vargas

**Affiliations:** ^1^Department of Systems Immunology and Braunschweig Integrated Centre of Systems Biology, Helmholtz Centre for Infection Research, Inhoffenstraße 7, 38124 Braunschweig, Germany; ^2^Institut für Geophysik und extraterrestrische Physik, Technische Universität Braunschweig, Mendelssohnstraße 3, 38106 Braunschweig, Germany; ^3^Institute for Biochemistry, Biotechnology and Bioinformatics, Braunschweig University of Technology, Braunschweig, 38106 Braunschweig, Germany

## Abstract

HIV is a widespread viral infection without cure. Drug treatment has transformed HIV disease into a treatable long-term infection. However, the appearance of mutations within the viral genome reduces the susceptibility of HIV to drugs. Therefore, a key goal is to extend the time until patients exhibit resistance to all existing drugs. Current HIV treatment guidelines seem poorly supported as practitioners have not achieved a consensus on the optimal time to initiate and to switch antiretroviral treatments. We contribute to this discussion with predictions derived from a mathematical model of HIV dynamics. Our results indicate that early therapy initiation (within 2 years postinfection) is critical to delay AIDS progression. For patients who have not received any therapy during the first 3 years postinfection, switch in response to virological failure may outperform proactive switching strategies. In case that proactive switching is opted, the switching time between therapies should not be larger than 100 days. Further clinical trials are needed to either confirm or falsify these predictions.

## 1. Introduction

According to the last report from UNAIDS in 2011 [1], 34 million people live with human immunodeficiency virus (HIV). Although the number of new cases of HIV infection is declining, the number of people living with HIV is increasing; therefore the problems of continuing treatment of chronic infection are of major importance in today's social medicine.

**Figure 1:**
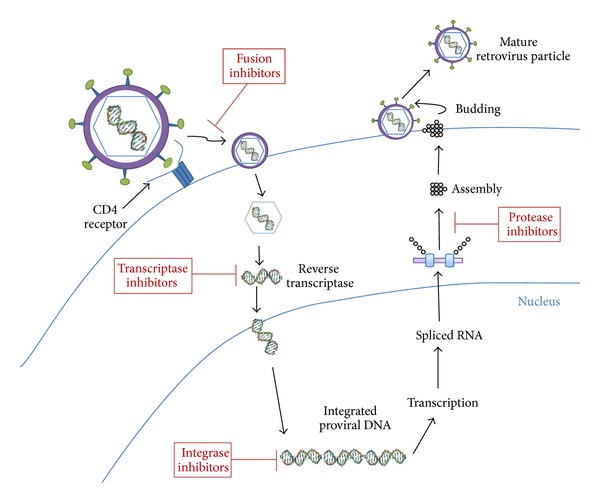
HIV infection cycle affected by the four distinct drug classes. The drug classes are shown in red boxes.

Nowadays, the drugs to treat HIV type 1 (HIV-1) infection belong to four distinct classes [2, 3]: reverse transcriptase inhibitors, protease inhibitors, integrase inhibitors, and fusion inhibitors (Figure 1). Currently, highly active antiretroviral therapies (HAART) generally comprise three different drugs to combat different parts of the HIV cycle. These therapies prevent immune deterioration, reduce morbidity and mortality, and prolong the life expectancy of people infected with HIV.

Nevertheless, HAART is not always successful. Many patients have long-term complications while others experience virological failure (inability to maintain HIV RNA levels below 50 copies/mL) [3]. In most cases, viral rebound is associated with the emergence of resistance-conferring mutations within the viral genome, resulting in reduced viral susceptibility to one or more of the drugs. This is related to the reverse transcription process of viral RNA into DNA, which is highly prone to errors, introducing on average one mutation for each viral genome transcribed [2].

The primary goal of the initial regimen proposed in the guidelines for the use of antiretroviral agents in HIV-1 infected adults and adolescents by Department of Health and Human Services (DHHS) [3] is to suppress viral replication to the maximum and to sustain this level of suppression as long as possible. Even though HAART can reduce the viral load in the blood by at least five orders of magnitude, ongoing low-level replication still occurs; hence, the risk of developing resistance is always present. Complete virus eradication by HAART does not appear to be achievable in the foreseeable future. Furthermore, antiretroviral guidelines [3] have not achieved a consensus on two fundamental questions in HIV treatment: (i) when to start antiretroviral treatment and (ii) when to change for a new antiretroviral treatment.

The standard procedure for therapy initiation is still a point of discussion in the expert panel over the last 20 years [3]. However, there is a general consensus that antiretroviral therapy should be initiated in all patients with a history of an AIDS-defining illness or when CD4+ T cell counts are less than 350 cells/mm^3^. It is strongly recommended to start therapy if the CD4+ T cell count is between 350 and 500 cells/mm^3^ and there is a recommendation with moderate urgency for patients with CD4+ T cell counts > 500 cells/mm^3^ (Table 1).

The expert panel [3] pointed out the absence of cohort studies that conclusively demonstrate a clinical benefit of HAART in patients with CD4+ T count > 350 cells/mm^3^. For some patients, the potential risks of short- or long-term drug-related complications and nonadherence to long-term therapy may offset possible benefits of earlier therapy initiation.

Computational biology may play an important role in evaluating the impact of the initiation time of HAART during HIV infection. Using a mathematical model, [4] suggested to initiate treatment in stages of the infection when the viral load can be easily controlled: in the acute phase of the infection when the viral load peaks and moderately in the asymptomatic phase. The very early phase and the AIDS phase are considered hardly controllable. Thus, authors in [4] argued in favour of an early but not immediate treatment initiation. The main drawback of this study is the lack of a mathematical model that is able to reproduce the whole disease trajectory, which limits the long-term assessments of treatment strategies. The observation that HAART timing has a strong impact on the disease outcome is supported by computational results by [5, 6]. However, these studies have similar drawbacks, as they do not envisage long-term dynamics (more than 8 years when AIDS may appear) and different treatment protocols.

For the second fundamental question in HIV treatment, when treatment should be alternated in response to virological failure, the answer is more complex [3, 7, 8]. One clinical goal is to delay the time until patients exhibit strains resistant to all existing regimens. There is a crucial trade-off between switching therapies. On the one hand, switching early carries the risk of poor adherence to a new drug regimen and prematurely exhausting the limited number of remaining salvage therapies. On the other hand, switching drugs too late allows the accumulation of mutations that leads to multidrug resistance [2, 3]. The most aggressive approach would be to change therapies for any repeated detectable viremia (e.g., two consecutive HIV RNA > 50 copies/mL after suppression). The most conservative strategy has been to allow detectable viremia up to an arbitrary level (e.g., 1000–500 copies/mL). This latter approach is called switch on virological failure [3].

Recently, Martinez-Picado et al. [9] suggested that switching between therapies can decrease the likelihood of viral resistance and prolong the pre-AIDS period. This hypothesis was supported by clinical trials [9] called* Switching Antiviral Therapy Combination against HIV* (SWATCH), which consists of two HAART regimens that are periodically alternated every 3 months. The alternation of 2 drug regimens could inhibit the emergence of highly resistant genotypes.

In this paper, we focus on the trade-off between the inhibition of long-term reservoirs and the promotion of resistant genotypes and thus help to precisely work out the advantages of early treatment. We also investigate how frequently antiretroviral regimens should be alternated in order to attain optimal proactive treatments.

## 2. Material and Methods

### 2.1. Mathematical Model

A typical HIV infection response consists of three stages: an initial acute infection, a long asymptomatic period, and a final increase in viral load with simultaneous collapse in healthy CD4+ T cell counts. The majority of existing mathematical models [4, 11–20] give a good representation of either the first two stages [11, 13, 21–24] or the last stage of the infection [25] but do not describe the three stages observed in HIV infection in a single framework. A mathematical model that is able to represent the typical HIV infection dynamics including all three stages was suggested by [26]. The model includes the possibility of full parameter variations, without losing the capability to describe the three stages.

**Figure 2:**
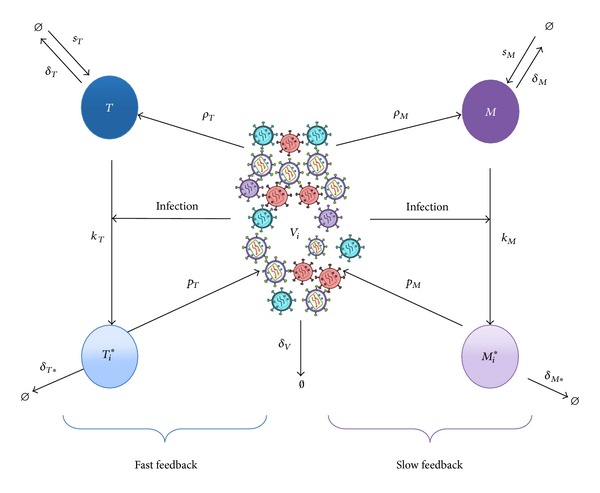
Nonlinear HIV model. *T* represents the uninfected CD4+ T cells, *T*_i_* represents the infected CD4+ T cells with the *i*th strain, *M* represents uninfected macrophages, *M*_i_* represents infected macrophages with the *i*th strain, *V*_*i*_ represents the *i*th strain, and *V*_*T*_ is the sum of all strains.

The results in [26] indicate that HIV infection can be considered as two feedback systems (Figure 2). One provides the fast dynamics presented in the early stages of infection as a result of the fast infection process of CD4+ T cells. The second feedback sustains a constant slow infection process in macrophages over the years accompanied by the long time survival conditions of macrophages.

Here, following the work by [8], we extend the mutation tree from [10] into a nonlinear model with mutations (1)–(5). Unlike existing models [4, 8, 11–20], our model is able to adequately represent the three stages of HIV infection and the dynamics of resistant genotypes when HAART treatment is introduced. The model is defined by the following set of differential equations:

(1) $$\begin{array}{c} \dot{T}=s_{T}+\frac{\rho_{T}}{C_{T}+V_{T}}TV_{T}-\overset{n}{\underset{i=1}{\sum }}k_{T}f_{i}\left(1-\eta_{\sigma ,i}^{T}\right)TV_{i}-\delta_{T}T, \end{array}$$

(2) $$\begin{array}{c} \dot{M}=s_{M}+\frac{\rho_{M}}{C_{M}+V_{T}}MV_{T}-\overset{n}{\underset{i=1}{\sum }}k_{M}f_{i}\left(1-\eta_{\sigma ,i}^{M}\right)MV_{i}-\delta_{M}M, \end{array}$$

(3) $$\begin{array}{c} {\dot{T}}_{i}^{*}=k_{T}f_{i}\left(1-\eta_{\sigma ,i}^{T}\right)TV_{i}+\overset{n}{\underset{j=1}{\sum }}\mu m_{i,j}V_{j}T-\delta_{T^{*}}T_{i}^{*}, \end{array}$$

(4) $$\begin{array}{c} {\dot{M}}_{i}^{*}=k_{M}f_{i}\left(1-\eta_{\sigma ,i}^{M}\right)MV_{i}+\overset{n}{\underset{j=1}{\sum }}\mu m_{i,j}V_{j}M-\delta_{M^{*}}M_{i}^{*}, \end{array}$$

(5) $$\begin{array}{c} {\dot{V}}_{i}=p_{T}f_{i}\left(1-\theta_{\sigma ,i}^{T}\right)T_{i}^{*}+p_{M}f_{i}\left(1-\theta_{\sigma ,i}^{M}\right)M_{i}^{*}-\delta_{V}V_{i}, \end{array}$$

where *T* represents the uninfected CD4+ T cells, *T*_i_* represents the infected CD4+ T cells with the *i*th strain (genetic variant or subtype of the virus), *M* represents uninfected macrophages, *M*_i_* represents infected macrophages with the *i*th strain, *V*_*i*_ represents the *i*th strain, and *V*_*T*_ is the sum of all *n* strains.

Parameters *s*_*T*_ and *s*_*M*_ represent the source terms of new CD4+ T cells and macrophages, respectively. HIV, as other pathogens, triggers the proliferation of immune cells. Homeostatic proliferation is modeled using a logistic growth model limited by viral load. This would allow convergence to a high percentage of the reservoirs (macrophages) being infected without allowing the total population to expand at unrealistic growth rates. Parameters *ρ*_*T*_ and *ρ*_*M*_ are the maximum proliferation rate for CD4+ T cells and macrophages, respectively. *C*_*T*_ and *C*_*M*_ represent the respective half-velocity constants.

The infection rate constant is represented with *k*_*T*_ for CD4+ T cells and *k*_*M*_ for macrophages. Viral proliferation is achieved in infected CD4+ T cells and infected macrophages with rate constants *p*_*T*_ and *p*_*M*_, respectively. These parameters depend on the fitness of the genotype (*f*_*i*_) and the therapy (*σ*) that is being used.

**Figure 3:**
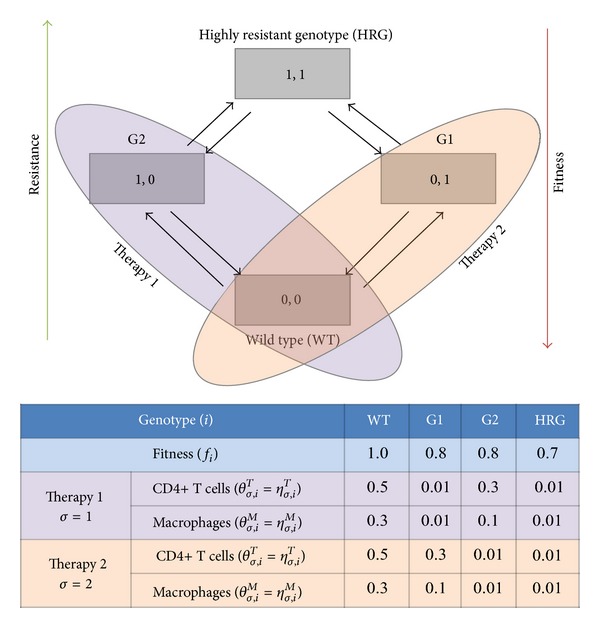
Four variant mutation trees. The wild type (*WT*) is susceptible to both therapies. Genotype 1 (*G1*) is susceptible to therapy 2 and genotype 2 (*G2*) is susceptible to therapy 1. The highly resistant genotype (*HRG*) is not affected by any therapy. Parameter values were taken from [10].

We consider two therapies (*σ* = 1,2) composed of reverse transcriptase inhibitors with effectiveness *η*_*σ*,*i*_^*T*^ for CD4+ T cells and *η*_*σ*,*i*_^*M*^ for macrophages and protease inhibitors with effectiveness *θ*_*σ*,*i*_^*T*^ for CD4+ T cells and *θ*_*σ*,*i*_^*M*^ for macrophages. Note that, based on clinical evidence [27], inhibitors are more effective in CD4+ T cells than in macrophages; this is considered by *η*_*σ*,*i*_^*T*^ > *η*_*σ*,*i*_^*M*^ and *θ*_*σ*,*i*_^*T*^ > *θ*_*σ*,*i*_^*M*^. The therapy parameter values used in this study are given in Figure 3.

The mutation rate is expressed by *μ*, and *m*_*i*,*j*_ ∈ {0,1} represents the genetic connections between genotypes. The degradation rates for the relevant species are *δ*_*T*_, *δ*_T*_, *δ*_*M*_, *δ*_M*_, and *δ*_*V*_. Parameter values are presented in Table 2.

The meaning of the different terms and parameters is illustrated in a block diagram (Figure 2). For example, the left-hand side of (1) defines the rate of change of the uninfected CD4+ T cells. The right-hand side defines how CD4+ T cells are altered during infection: a constant production of CD4+ T cells is expressed by *s*_*T*_. CD4+ T cell depletion is proportional to the death rate *δ*_*T*_ and the current number of healthy CD4+ T cells. The cell proliferation is proportional to the number of healthy cells and the total viral load *V*_*T*_, which represents the sum over all strains (*n*). The infection is described by the sum term on the right-hand side of (1) and is also proportional to the existing number of uninfected cells and the strains *V*_*i*_. Each strain has its own infection rate constant *k*_*T*_ *f*_*i*_(1 − *η*_*σ*,*i*_^*T*^), which is why we have to sum over index *i*. The other equations may be understood in a similar fashion.

### 2.2. Viral Mutation Tree

Nowadays, it is considered critical to take viral mutation into account during the development of treatment strategies. The process of reverse transcription is extremely error-prone and it is during this step that mutations can occur. High levels of resistance can be produced by substitutions of a single amino acid [3]. For instance, when lamivudine is used as a single agent, resistant strains will appear in a few weeks [2]. This is the reason why monotherapy has been discontinued and HAART is composed of at least three different drugs. As a result, multiple mutations are required for resistance to occur to all drugs in one regimen.

As a simple motivating example, we consider the mutation tree with 4 variants and 2 possible antiretroviral treatments proposed in [10]. The wild type genotype (*WT*) would be the most prolific variant in the absence of any drugs (Figure 3). However, it is also the variant that all drug combinations have been designed to combat and therefore is susceptible to all therapies. The wild type genotype (*WT*) can mutate to either genotype 1 (*G1*) which is susceptible to therapy 2 or genotype 2 (*G2*) which is susceptible to therapy 1. After mutations, the highly resistant genotype (*HRG*) is a genotype with a low proliferation rate, but resistant to all drug therapies.

### 2.3. Therapy

The DHHS panel [3] recommends therapies with two nucleoside analogues and either protease inhibitors or nucleoside reverse transcriptase inhibitors. The combination of these drugs is crucial in controlling the development of resistance. For simulation purposes, we consider the common clinical strategy suggested by the DHHS panel in [3], which recommends change therapy when virological failure is presented, that is, switch on virological failure (SVF): introduce a new regimen if there is detectable viremia (HIV RNA > 1,000 copies/mL) and drug-resistant genotypes are identified. We also consider the SWATCH strategy proposed in [9]. The rationale behind this strategy is that one could preempt virologic rebound and reduce accumulating drug-resistant genotypes by alternating treatments. This strategy is implemented as follows:

 
*SWATCH*: alternate between two regimens every 3 months.

### 2.4. Monte Carlo Simulations

**Figure 4:**
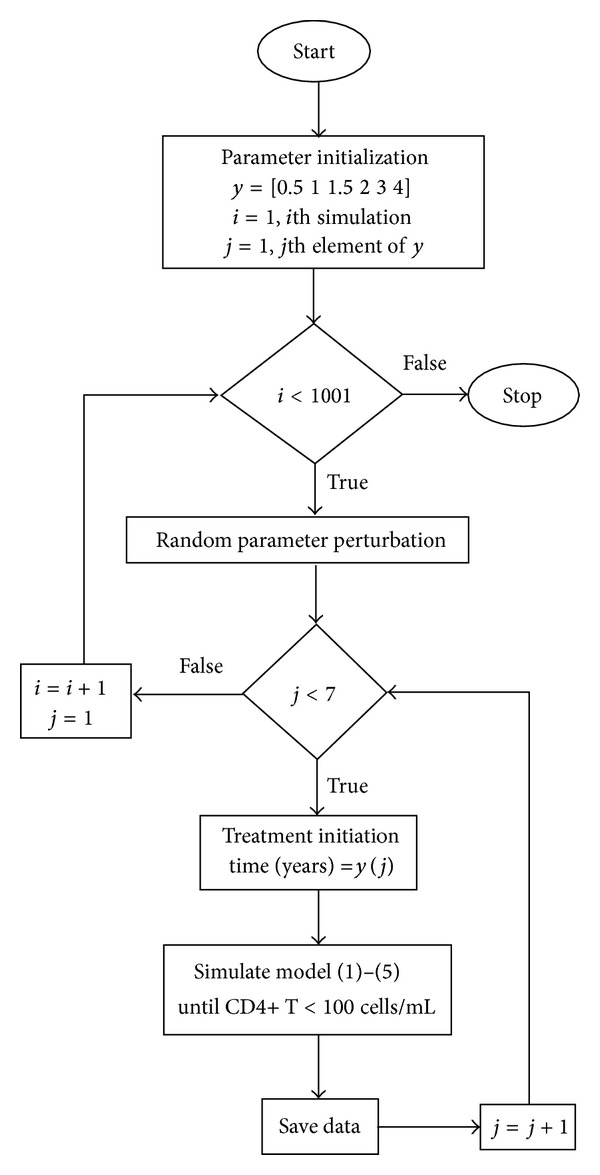
Monte Carlo simulation algorithm. The Simulation box includes the simulation of the four-genotype mutation model under a certain treatment. For each of the six times of treatment initiation, the simulation will be repeated 1000 times and the data can be analysed.

As the interpatient variability and the extreme error sensitivity of the HIV replication process are nonnegligible in HIV treatment, we perform Monte Carlo (MC) simulations to analyse treatment strategies in stochastic environments [30]. Our MC algorithm consists of repeated random sampling, providing numerical results that can be interpreted with statistical methods. To this end, we consider 1000 repeated simulations with randomly perturbed parameters (normal distribution and 30% deviation from the nominal parameter values in Table 2). The described MC simulations without therapy, with SVF, and SWATCH treatments were carried out at six different initiation times *t*_*i*_ (0.5, 1, 1.5, 2, 3, and 4 years). The differential equations (1)–(5) are solved with the toolbox *ode*45 from the MATLAB library. We record the year when immunological failure appears (*t*_*f*_), that is, when CD4+ T cells sink under 200 copies/mm^3^. *N*_*p*_ represents the number of cases where no immunological failure occurs during 30 years. The MC procedure is illustrated as a flow diagram in Figure 4. The analysis of the MC simulation results is based on a two-way ANOVA test. The significance is identified as *P* value <0.05; the data are further analyzed by a two-way *t*-test and a Bonferroni posttest.

## 3. Results and Discussion

For the scenario with no treatment, we obtain an average time of immunological failure of 8.5 years postinfection from our MC simulations. In addition, 99% of the simulated cases may progress to AIDS at different time scales. These results are consistent with clinical observations in [31], suggesting that model (1)–(5) can adequately represent the basic features of HIV infection.

### 3.1. Treatment Initiation

**Figure 5:**
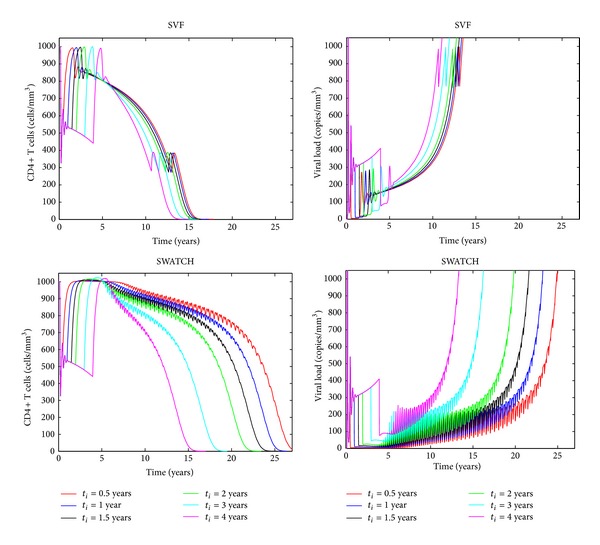
SVF and SWATCH treatment strategies. CD4+ T cells and viral load are displayed for different initiation times (*t*_*i*_). The top two panels show simulation results for the SVF strategy and the bottom panels show the results for the SWATCH strategy. Deterministic simulations are based on nominal parameter values from Table 2.

The treatment strategies SVF and SWATCH (Figure 5) were implemented in model (1)–(5) using different initiation times *t*_*i*_ (0.5, 1, 1.5, 2, 3, and 4 years) and parameter values as presented in Table 2. During the first 5 years, we observe that both SVF and SWATCH satisfy the levels required for healthy immunological responses (CD4+ T > 500 cells/mm^3^) and decrease the viral load to undetectable levels (<50 copies/mL) as suggested in [3].

For both treatment strategies, our results support the hypothesis that an early treatment initiation is more beneficial than a late start of the therapy. However, simulation results for the SVF strategy (Figure 5) suggest that there is only little improvement for a very early treatment initiation (<1 year).

If a second treatment is introduced, a second virological failure may appear, faster than the first one, progressing to AIDS (Figure 5). This is consistent with the observation in [32] that persistent low-level viremia and long-term reservoirs promote a second virological failure.

The clinical trial SWATCH [9] suggested an alternative solution to minimize HIV resistance mutation by alternating between two regimens every three months while viral load is suppressed. In our simulations, the alternation is visible in form of the high-frequency oscillations. For early initiation treatment (<2 years), the results in Figure 5 indicate that the SWATCH approach is clearly superior to SVF because the time for immunological failure may occur 10 years later compared to the SVF strategy. However, SWATCH is more sensitive to the initiation time. For instance, when *t*_*i*_ = 4 years, SWATCH and SVF may show similar performance. We conclude that SWATCH treatment may provide significant extension of the time to virological failure only if the treatment is initiated before the third year postinfection.

While the results above were obtained for one particular parameter set, we now consider the nonlinearity of the problem and the corresponding sensitivity to parameter variations by using an MC approach with 1000 random samples (Table 3). In comparison to the MC simulations without treatment, we note in Table 3 that the number of cases without immunological failure (*N*_*p*_) increases substantially (approximately 30%). For instance, when *t*_*i*_ = 0.5, MC simulations suggest that SVF may achieve 29.8% cases without immunological failure while SWATCH may achieve up to 46.6%. Furthermore, the appearance of virological failure is prolonged approximately by 4–7 years (*P* value ≤0.05).

**Figure 6:**
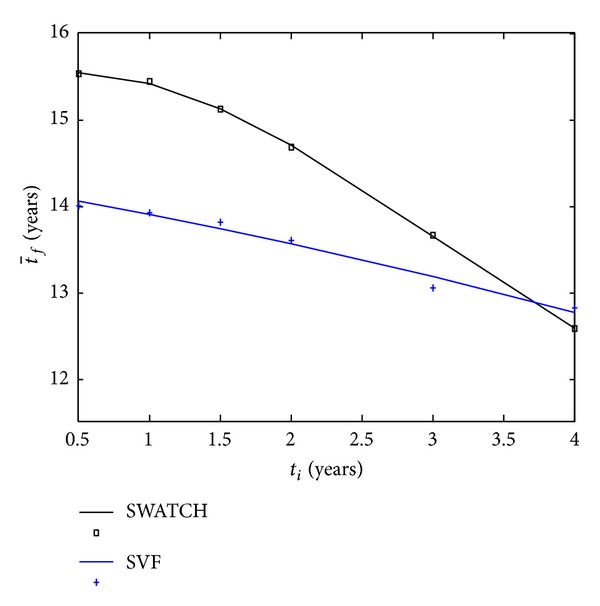
Monte Carlo simulation outcome for SVF (+) and SWATCH (□) strategies. Averaged time of immunological failure (${\overset{-}{t}}_{f}$) based on 1000 random samples is plotted against different initiation times (*t*_*i*_). A parabolic function was fitted to the SWATCH data (solid black line) and a linear function to the SVF data (solid blue line).

Our studies support the hypothesis that an early intervention has significant positive impact on postponing the progression to AIDS.  Figure 6 shows the average time when immunological failure occurs (${\bar{t}}_{f}$) as a function of the initial time (*t*_*i*_). We can see that SVF performance decreases almost linearly with respect to the initial time, while SWATCH shows approximately a parabolic behaviour. Note that the SWATCH strategy can outperform the SVF strategy only when therapy is initiated before the second year postinfection (*P* value ≤0.05). Therefore, it is not recommended to use the SWATCH strategy on patients who have not initiated treatment during 3 years postinfection.

Our results lead us to recommend avoiding treatment initiation after 2 years postinfection. In addition, for patients who have not received any treatment within 3 years or more postinfection, the SVF strategy is a better alternative since the advantage of SWATCH is fading (Figure 6) while the risk of long-term drug toxication could be smaller with the SVF strategy.

### 3.2. Alternating between Treatments

Computational studies [7, 8, 10] and clinical trials [9] suggest that a proactive alternating strategy like SWATCH may yield promising results. However, not enough work has been done to analyse the synergistic effect of initiation time and period of alternation between treatments.

**Figure 7:**
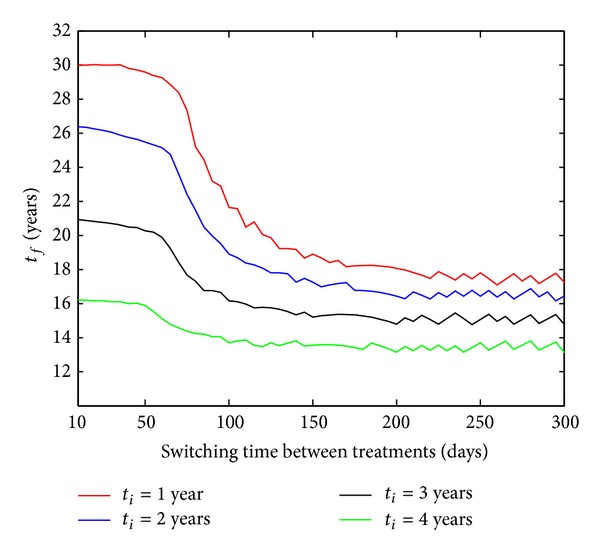
Optimal switching between regimens. Time of immunological failure (*t*_*f*_) with respect to switching time for different initiation times (*t*_*i*_).

Here, we ran model (1)–(5) with different switching and different initiation times. The first observation is that fast switching times between regimens yield longer periods without immunological failure (Figure 7). In addition, for switching times larger than 150 days, simulation trajectories reached a steady state, meaning that long periods between treatment switches should be avoided. An aspect that is not covered here, however, is that fast switching between regimens (<60 days) could increase drug toxicity and may lead to bad adherence to therapy. Further studies (pharmacokinetics/pharmacodynamics) are needed to include such issues to develop a comprehensive management concept.

A second important conclusion from Figure 7 is that the SWATCH strategy seems to perform poorly if the treatment was initiated 2 years postinfection. To attain a high effectiveness of the treatment, the switching frequency between antiretroviral regimens may need to be increased depending on the delay of therapy initiation. Considering *t*_*i*_ = 3 or *t*_*i*_ = 4 years, our results suggest that very short switching times about 10–60 days would be required to obtain the same performance as if the therapy was started earlier. Consistent with the results in Figure 6, this investigation leads to the conclusion that the SWATCH strategy should be initiated within 2 years postinfection to achieve responses superior to common clinical approaches (SVF).

Our results reveal the importance of early therapy to delay the AIDS progression. There are hints that an early intervention could improve the patient healing process. The recent clinical results by [33] showed the possibility of a mechanistic cure for patients who are treated immediately after the infection. A mechanistic cure means a permanent viral suppression in the absence of therapy to levels that prevent immunodeficiency and transmission. However, it was not possible to simulate this behaviour using model (1)–(5). This is likely because the model is based on ODEs, implying that populations arbitrarily close to zero can recover. Discrete approaches could bring new insights into this field.

## 4. Conclusions

From our studies, we conclude that antiretroviral treatment strategies initiated after 2 years postinfection are not beneficial to extend the time to progression to AIDS.

Another significant result is that the SWATCH strategy outperforms SVF only when therapies are initiated within 2 years postinfection and switching periods for SWATCH strategy are less than 90 days. Large switching periods between regimens (>100 days) should be avoided during the application of SWATCH.

This work is a step forward for defining criteria for when to initiate and alternate therapy. Future work will be directed to the experimental evaluation of the presented results.

## Figures and Tables

**Table 1:** Panel recommendations for therapy initiation.

		Urgency of initiation
CD4+ T cell count	<350 cells/mm^3^	Strong I
	350–500 cells/mm^3^	Strong II
	>500 cells/mm^3^	Moderate III

Transmission risk	Perinatal	Strong I
	Heterosexual	Strong I
	Other	Strong III

The urgency is rated by validity: I: data from randomized trials, II: data from well-designed nonrandomized trials or observational cohort studies with long-term clinical outcomes, III: expert opinion [3].

**Table 2:** Parameter values for (1)–(5).

Parameter	Units	Nominal value	Source
*s*_*T*_	Cells/mm^3^ day	10	[28]
s_M_	Cells/mm^3^ day	0.15	[28]
k_T_	mm^3^/day copies	4.57 × 10^−5^	[28]
k_M_	mm^3^/day copies	4.33 × 10^−8^	[26]
p_T_	Copies/cell day	38	[29]
p_M_	Copies/cell day	35	[29]
δ_T_	Day^−1^	0.01	[29]
δ_T_*	Day^−1^	0.4	[29]
δ_M_	Day^−1^	1 × 10^−3^	[26]
δ_M_*	Day^−1^	1 × 10^−3^	[26]
δ_V_	Day^−1^	2.4	[29]
ρ_T_	Day^−1^	0.01	[26]
ρ_M_	Day^−1^	0.003	[26]
C_T_	Copies/mm^3^	300	[26]
C_M_	Copies/mm^3^	220	[26]

**Table:** (a) SVF

*t*_*i*_ (years)	${\bar{t}}_{f}\pm \bar{\sigma }$ (years)	*N*_*p*_
0.5	14.01 ± 4.34	298
1	13.93 ± 4.39	292
1.5	13.82 ± 4.43	284
2	13.61 ± 4.38	277
3*	13.06 ± 4.08	259
4*	12.83 ± 4.55	253

**Table:** (b) SWATCH

*t*_*i*_ (years)	${\bar{t}}_{f}\pm \bar{\sigma }$ (years)	*N*_*p*_
0.5	15.54 ± 4.79	466
1	15.45 ± 4.81	454
1.5	15.13 ± 4.79	442
2*	14.69 ± 4.68	427
3*	13.67 ± 4.38	386
4*	12.59 ± 3.84	336

${\bar{t}}_{f}$ is the average year when immunological failure occurs for the 1000 random samples and the respective standard deviation $\bar{\sigma }$. *N*_*p*_ is the number of cases that do not experience immunological failure after 30 years postinfection. *Star represents a statistically significant difference with respect to the group of *t*_*i*_ = 0.5 years (*P*-value ≤ 0.05). There is a significant difference between SWATCH and SVF for all the groups when *t*_*i*_ ≤ 2.
